# Current practice in mammographic imaging of the augmented breast in Australia

**DOI:** 10.1002/jmrs.374

**Published:** 2020-01-24

**Authors:** Jacquelyn R O'Keefe, Jenny Maree Wilkinson, Kelly Maree Spuur

**Affiliations:** ^1^ Faculty of Science, School of Dentistry & Health Sciences Charles Sturt University Wagga Wagga New South Wales Australia

**Keywords:** breast imaging, breast augmentation, Eklund technique, mammography, breast cancer

## Abstract

**Aim:**

This study seeks to document the imaging series used in contemporary Australian practice for imaging the augmented breast, with a secondary focus on differences in practice and opinion between BreastScreen Australia and diagnostic imaging services.

**Methods:**

A SurveyMonkey link was distributed through the Australian Society of Medical Imaging and Radiation Therapy (ASMIRT) and was assessable during December 2017 and January 2018. The questionnaire investigated: years of experience, facility type and location, image acquisition systems, appointment times, patients imaged per week, technique and imaging series used, use of limited compression views, rationale for variation in imaging series and the use of ultrasound. Descriptive statistics were produced for all variables with chi‐squared tests used for comparisons between categorical variables.

**Results:**

The most frequently used series was the eight‐image Eklund ID technique 64% and 59% (submuscular) and 68% and 58% (subglandular) for BSA and diagnostic services, respectively. Eighteen different combinations of projections were reported with eight combinations common to both subglandular and submuscular imaging. The majority of participants attributed imaging series preferences to dose reduction and radiologist preference.

**Conclusion:**

This research has demonstrated varied approaches to the routine imaging of women with breast implants and identified the need for the establishment of dedicated evidence‐based imaging protocols to ensure that regardless of which setting a woman attends that they receive standardised imaging with minimal dose and maximum breast coverage. This is a reassurance that is not applicable to current practice.

## Graphical abstract

This study documents the imaging series used in contemporary Australian practice for imaging the augmented breast. Eighteen different combinations of projections were reported to be used for routine imaging with eight combinations common to both subglandular and submuscular imaging; the most frequently cited series was the eight‐image Eklund ID technique. The results demonstrate varied approaches to the routine imaging of women with breast implants identifying the need for the establishment of dedicated evidence‐based protocols for imaging of the augmented breast.

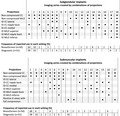

## Introduction

Mammography is the gold standard for the detection, analysis and diagnosis of breast abnormalities including breast cancer. 1 Breast cancer is the second most common malignancy worldwide and is ranked as the most common cause of cancer‐related death in less developed countries and second to lung cancer in developed countries. 2 In Australia, there is a 1 in 8 risks that a woman will develop breast cancer before the age of 85. 3 Mammography is undertaken through the Australian government funded BreastScreen Australia (BSA) programme and in diagnostic private practice facilities. 4, 5 In both settings, the craniocaudal (CC) view and the mediolateral oblique view (MLO) are routinely performed as these projections afford the greatest coverage of breast tissue. 6

In 2016, breast augmentation was ranked the number one most commonly undertaken cosmetic surgical procedures worldwide with 1.6 million surgeries reported. 7 Augmentation is also recognised as the most common cosmetic surgical procedure in Australia, with approximately 17,000 surgeries performed in 2016.^7^ Indications for augmentation can generally be divided between cosmetic enhancement, the correction of congenital and developmental deformities and reconstruction.^8^, ^9^ Breast implants however present a technical challenge with respect to imaging due to their radiopacity, which obscures breast tissue and reduces the sensitivity of imaging.^10^, ^11^, ^12^ The favoured surgical placement of the implant is anterior to the pectoral muscle (subglandular); however, submuscular insertion, behind the pectoral muscle, is also common.^13^ Implant placement is typically dependent on the surgeon's preference and the desired cosmetic outcome and while submuscular placement reduces the overall impact on imaging, there is still a requirement for a specialised technique to appropriately image the breast.^14^ There is no conclusive evidence that the reduced sensitivity of mammographic imaging due to the presence of implants leads to late cancer detection or poorer prognosis than for women with non‐augmented breasts.^15^, ^16^, ^17^

To improve imaging of the augmented breast, a dedicated method of implant imaging was devised in 1988, by Eklund et al., and is most commonly referred to as the implant displacement (ID) technique (also known as the Eklund technique).^18^ The ID technique has been widely adopted for routine imaging of the augmented breast 6, 19, 20, 21, 22, 23 and involves pushing the implant superiorly and posteriorly towards the chest wall while the available breast tissue is pulled anteriorly and compressed.^18^ Typically, ID imaging will not be attempted before completion of a CC and MLO imaging series with very limited to no compression. This is in order to determine implant integrity prior to compression for ID imaging.^24^ Non‐compressed views with the implant in place can also allow the radiographer to gauge the amount of tissue available for compression, thus assisting positioning. The imaging series described by Eklund et al. (Eklund series) mandates a series of eight images, which includes both limited compression (also termed non‐compression views) and ID images in the CC and MLO projection for both breasts.^18^

The most common complication of breast augmentation is capsular contraction caused by an inflammatory reaction and resulting in fibrosis. 25 Capsular contracture, or encapsulation of the breast implant, results in hardening and deformity of the implant 26 and, in severe cases, limits the degree of ID, thus reducing the ability to adequately demonstrate breast tissue.^27^ To manage this, the addition of a lateral projection is advocated by many sources including Eklund et al.^15^, ^18^, ^23^, ^28^ These additional views in bilateral breast imaging result in a ten‐image series. There is no supporting evidence to suggest true laterals provide sufficient coverage of the breast tissue in these cases.^23^

In 1992, Rickard et al. published on a new imaging series based upon the ID technique to provide a more thorough examination of the augmented breast.^29^ This sixteen‐image series (herein called Rickard ID series) is known to have been in use prior to the implementation of full field digital mammography (FFDM) and digital breast tomosynthesis (DBT) in Australia 30 and recommends an additional four ID views per breast to further demonstrate breast tissue medial and lateral to, and superior and inferior to the implant in the CC and MLO projection, respectively.^29^

In addition to an increase in radiation dose due to the increased number of routine projections when utilising the ID technique, Smathers et al. (2007) identified that patients also receive higher doses of radiation due to the increased size of their breasts.^31^ Increased breast size is of particular concern in limited compression views. While some authors assumed that the mammography dose in the presence of breast implants was doubled 32, 33, Smathers et al. demonstrated 3.1 times higher radiation dose received using the eight‐image Eklund series when compared to conventional four‐image mammography for the non‐augmented breast.^31^

There is a lack of guidelines for the imaging of breast implant patients in Australia. While the ID method is encouraged, no specific implant imaging protocol (Eklund ID series, Rickard ID series or other) is listed in BreastScreen Australia's National Accreditation Standards (BSA NAS) or in the Royal Australian and New Zealand College of Radiologist (RANZCR) Mammography Quality Control Manual.^6^, ^20^ Review of the literature for the mammographic assessment of patients with breast implants provides evidence of the wider issue of non‐standardised approaches to imaging on an international level. Educational texts and the accreditation standards of various countries describing imaging of the augmented breast are widely variable, and the imaging series described demonstrate this disparity with between a 6‐ and 16‐image series being reported.^6^, ^18^, ^19^, ^21^, ^22^, ^23^, ^29^, ^30^, ^34^, ^35^, ^36^, ^37^ There is no evidence in the literature to support a particular imaging series as the gold standard for the routine imaging series for a patient with breast implants and there is no clear evidence of which series is being routinely undertaken in Australia.

This study investigated the current practice with respect to imaging of augmented breasts in the diagnostic and BreastScreen Australia (BSA) setting. It is unknown if the approach to imaging the augmented breast in each setting is in common with each other.

## Method

An online questionnaire was developed to gather information related to current practices for imaging of augmented breasts with the online platform, SurveyMonkey^®^ (https://www.surveymonkey.com), used to manage the questionnaire and collect responses. The questionnaire was designed to gather information on routine imaging practices in diagnostic and BreastScreen settings. Questions investigated were as follows: years of experience, facility type and location, image acquisition systems, appointment times, patients imaged per week, the technique and imaging series used, use of limited compression views, rationale for variation in imaging series and the use of ultrasound.

To understand imaging behaviours, respondents were asked to report use of one or more of the following when performing routine imaging of those with implants, those with submuscular implants, and when using tomosynthesis. The views were as follows: non‐compressed CC and MLO, implant displaced CC – lateral, nipple back or medial; implant displaced MLO – superior, nipple back or inferior/inframammary angle (IMA). In addition for submuscular implants, an option ‘routine 4 views with full compression’ was included.

The specific questions were devised based on the literature and 27 years of clinical experience in both diagnostic and screening mammography of one of the authors (KS). The questionnaire was edited for clarity through the Charles Sturt University (CSU) Spatial Data Analysis Network (SPAN).

A SurveyMonkey link was distributed through the Australian Society of Medical Imaging and Radiation Therapy (ASMIRT) e‐newsletter and was accessible during December 2017 and January 2018. The ASMIRT has 8091 members comprised of radiographers, radiation therapists, sonographers and mammographic technologists.^38^ The survey invitation was directed at members with experience in mammographic imaging only.

The absolute number of medical radiation science professionals who perform mammographic imaging in Australia is unknown as there is no official monitoring of this statistic by the Australian Health Practitioner Regulation Agency (AHPRA) or the professional body, ASMIRT. Some insight into population size may be gained considering the number of ASMIRT members who hold a Certificate of Clinical Proficiency in Mammography (CCPM); however, medical radiation science professionals practicing mammography in Australia are not required by AHPRA to acquire the CCPM. Currently, 712 ASMIRT members hold the CCPM; however, there have been 1532 CCPM awarded since 1998 (ASMIRT, correspondence 2019). As the survey encouraged participation for members with mammography experience past or present, 1532 could be considered closer to the true population of Australian medical radiation science professionals who have undertaken mammography. An ideal sample size of 308 was calculated, with a confidence level of 95%.^39^

Data obtained through the questionnaire were analysed using SPSS, Version 23 (IBM Corp. Released 2015. IBM SPSS Statistics for Windows, Version 23.0. Armonk, NY: IBM Corp.). Descriptive statistics were produced for all variables with chi‐squared tests used for comparisons between categorical variables with *P* < 0.05 deemed to be statistically significant. Free‐text responses were analysed thematically.

Ethics approval for this study was granted by the CSU Human Research Ethics Committee (HREC) (Approval number: 400/2017/34).

## Results

### Demographics

One hundred and eleven (111) responses were received during the collection period with 21 responses subsequently deemed incomplete and excluded from further analysis.

Of the 90 completed surveys, 40% (*n* = 36) worked for a diagnostic service, 37% (*n* = 33) worked for BSA and 23% (*n* = 21) worked in both settings. Overall the highest portion of respondents was from Queensland (30%) and New South Wales (26%) with smaller percentages from the other States/Territories, except the Northern Territory where there were no respondents. The majority of respondents (69%) worked in metropolitan settings with populations greater than 100,000^40^. At least 50% of respondents from each practice type had 16 or more years’ experience (Table 1). While there was some variation between location for the State/Territory and years of experience based on practice type, the number of responses was too small to draw conclusions about whether differences were statistically significant.

**Table 1:** Summary of respondent demographic information.

	All respondents (*n* = 90)	Type of mammography practice		
		Diagnostic (*n* = 36)	BreastScreen (*n* = 33)	Both (*n* = 21)
Years of experience				
1 year or less	2 (2%)	2 (6%)	0	0
2–5 years	13 (14%)	7 (19%)	2 (6%)	4 (19%)
6–10 years	10 (11%)	3 (8%)	4 (12%)	3 (14%)
11–15 years	13 (14%)	5 (14%)	6 (18%)	2 (10%)
16–20 years	20 (22%)	10 (28%)	9 (27%)	1 (5%)
20 or more years	32 (36%)	9 (25%)	12 (36%)	11 (52%)
Practice location (based on population size)*				
Metropolitan (> 100,000)	61 (69%)	23 (64%)	25 (78%)	13 (62%)
Regional (20,000–100,000	25 (28%)	12 (33%)	6 (18%)	7 (33%)
Rural (<20,000)	3 (3%)	1 (3%)	1 (5%)	3 (3%)
State or Territory where practice is located*				
Victoria	16 (18%)	3 (8%)	5 (15%)	8 (40%)
New South Wales	23 (26%)	6 (17%)	11 (33%)	6 (30%)
Queensland	27 (30%)	18 (50%)	7 (21%)	2 (10%)
Northern territory	0	0	0	0
Australian Capital Territory	2 (2%)	2 (6%)	0	0
South Australia	7 (8%)	4 (11%)	3 (9%)	0
Western Australia	12 (14%)	3 (8%)	5 (15%)	4 (20%)
Tasmania	2 (2%)	0	2 (6%)	0

*Two respondents did not provide a response questions about location and 1 to state; category totals may not be exactly 100% due to rounding.

The image acquisition systems used by respondents working for BSA were FFDM alone (56%) or FFDM and tomosynthesis (41%); the remaining 2 respondents had used either a combination of film screen, computed radiography (CR) and FFDM, or CR and tomosynthesis. In contrast for those working in diagnostic settings, the most frequent system, or combination of systems, being used were FFDM and tomosynthesis (46%), FFDM only or tomosynthesis only (both 22%); remaining respondents used a mix of film screen, CR, FFDM and tomosynthesis.

For those working in BSA settings, most (51%) imaged 2–4 women with implants per week; 23% reported imaging 0 or 1 woman per week and 13% reported imaging each of 4–6 or greater than 6 women per week. Those working in diagnostic settings reported imaging fewer women with implants per week with 55% reporting imaging 0–1 woman and 32% imaging 2–4 women. In this group, a small number (*n* = 6, 11%) reported imaging >6 women per week. Chi‐squared analysis showed that there was a statistically significant difference between the two groups (*χ* ^2^ = 13.898, df = 3, *P* = 0.003).

Most respondents (95%) working in BSA settings reported that the average appointment time for women without implants was 15 min or less; however, for women with implants there was an increase in average appointment time with 39% of respondents reporting an average time of 20 min and 26% reporting an average time of 30 min or more. In contrast, those in diagnostic settings reported longer average appointment times for women with or without implants with the most frequent response being 30 min (57% for those with no implants, 59% for those with implants).

### Imaging behaviour

The Eklund ID technique was reported as always used by 57% of BSA respondents; 15% reports use sometimes, 2% rarely and 11% did not use this technique. Similarly, 50% of those in diagnostic settings always used the Eklund ID technique, 19% sometimes, 8% rarely and 10% did not use the technique. The remaining respondents in each setting (15% for BSA, 14% for diagnostic) were unsure whether they used this technique. When asked why the Eklund technique was not used, the highest frequency single response for those at BSA was ‘unknown’ (23%) followed by ‘dose reduction’ (13%). The remaining respondents selected a combination of the item response options which included radiologist preference, dose reduction, not needed due to new technology (FFDM) and not needed due to new technology (tomosynthesis) with combinations which included radiologist preference and or dose reduction selected by 51% of respondents. Similarly, for those in diagnostic setting ‘unknown’ (16%) was the highest frequency single response with remaining responses being a combination of the available response options. The majority (73%) were combinations which included radiologist preference &/or dose reduction. The routine use of non‐compressed views prior to ID imaging was reported by the majority of respondents; 85% for BSA and 83% for diagnostic.

Reflective of the differing purposes of breast screening and diagnostic imaging, the routine use of ultrasound as an adjunct to routine implant imagining proved rare amongst BSA respondents with 80% indicating it is never used in their service; whilst the majority of diagnostic respondents (89%) indicated ultrasound is always or usually scheduled for patients.

#### Number of Images

Respondents were asked on a range of 4–16 how many images are routinely taken for patients with breast implants. For BSA, 91% of respondents reported taking 8 images, while 6% took 10 images and 4% ‘other’. While the majority (79%) of those in diagnostic settings also took 8 images, there was greater variability with 10% taking 10 images, 6% taken 4 images and 2% each taking 6, 12 or 16 images.

#### Breast implant image series

**Figure 1:**
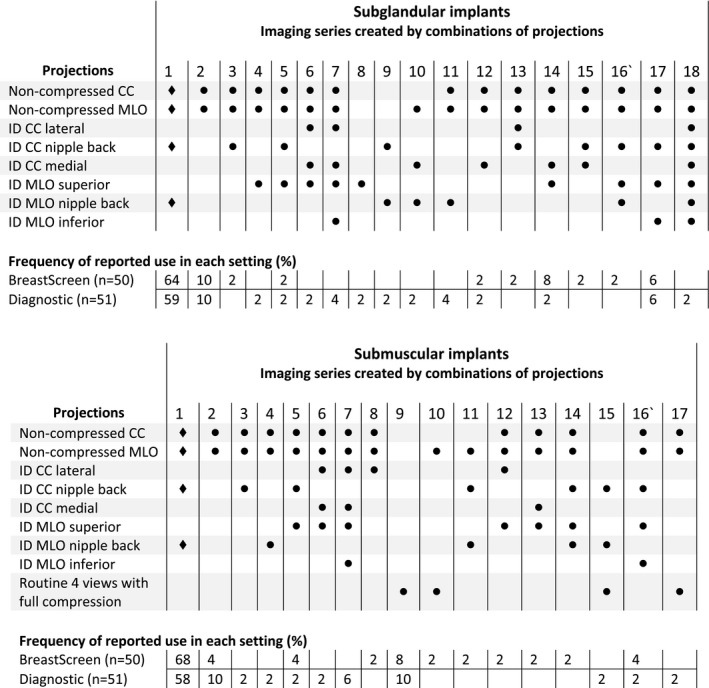
Image series created by combinations of projections for subglandular and submuscular implants.

Respondents were asked which of 8 projections were used when imaging patients with breast implants; this resulted in a total of 18 different combinations (Fig. 1). For both BSA and diagnostic settings, the most frequently used series was the eight‐image Eklund ID technique (64% for BSA and 59% for diagnostic settings). Although there was a range of image series reported, most were reported by only 1 or 2 respondents. Responses specifically in relation to imaging of submuscular implants were similar to those for implants with a range of combinations of projection used (Fig. 1). The Eklund technique was the most frequently used series in both groups (68% of BSA and 58% for diagnostic settings). Eight combinations (series 1–8 in Fig. 1) were common to both subglandular and submuscular imaging.

#### Use of tomosynthesis for imaging

In response to a question about the standard views for a patient with breast implants when using tomosynthesis, 78% of those in BSA settings and 41% of those in diagnostic settings indicated that they did not use tomosynthesis. Of the remaining respondents, again there were 5 different combinations of views used. While the number of respondents using tomosynthesis in BSA settings was small (*n* = 10), 50% used a combination of implant displaced CC and MLO nipple back views. For those in diagnostic settings, 11 different combinations were used with 31% using the eight‐image Eklund series. Other combinations were used by 4 or fewer respondents.

#### Free‐text responses

The majority of free‐text responses were utilised to rationalise the imaging series used, or to provide personal justification for the response given. Common themes included use of tomosynthesis at assessment in BreastScreen, advocating for true lateral imaging in cases of encapsulation or difficulty with ID views, or practice variations between the combined use of tomosynthesis and 2D imaging within a single series.

## Discussion

The results of this study demonstrate a range of practices in Australian radiographers working in BreastScreen and diagnostic settings. Reporting of appointment times reflected standard differences between the two settings reflective of the different appointment purposes. Diagnostic appointments are typically longer for women with and without implants to accommodate potential work up views and adjunct imaging. This is in contrast to screening where only routine imaging is performed and where needed, work up views and adjunct imagining is undertaken at a subsequent assessment appointment.

With respect to imaging practices, this study has identified a possible lack of consistent terminology. Whereas every ID image is by default undertaken using the Eklund technique, the Eklund ID technique was reported by BSA and diagnostic respondents, respectively, as always used by only 57% and 50%, with 15% and 14% being unsure if they utilised this technique at all. These responses appear to reveal the use of different imaging terminologies, which may reflect workplace conventions, differing educational approaches or may simply be a move away from eponyms.

As per imaging of the non‐augmented breast, the mammographic imaging protocol for women with augmented breasts should be standardised, evidence‐based and applicable to both the screening and diagnostic settings, and importantly, integrated into professional guidelines and accreditation standards. In the Australian healthcare setting, women should be reassured of receiving the same standard of imaging regardless of the facility to which they present. The results of this study demonstrate that the eight‐view ID technique (Eklund series) including non‐compressed CC, non‐compressed MLO, ID CC and ID MLO was the most frequently selected image series for both subglandular and submuscular implant imaging (BSA 64% and diagnostic 59%; BSA 68% and diagnostic 58%). However, of great concern is that the remaining respondents reported 18 and 17 different combinations of views forming a variety of imaging series being used to image subglandular and submuscular implants, respectively.

Protocols need to demonstrate some flexibility to accommodate for variability in patient presentation, such as encapsulation or minimal tissue volume. However, the wide variation in the imaging series reported cannot be explained by the need to accommodate patient presentation alone. Protocols such as those developed for the NHSBSP, which take into account patient history, implant type (saline or silicone) and position, provide a contemporary framework on which to base any future Australian protocols.^23^

In terms of imaging technique, the order of projections should be pre‐set; the NHSBSP advocates initial use of the non‐compressed MLO projection to show implant position and assist in the planning of subsequent projections.^23^ This appears to be a historically consistent practice. Non‐compressed views should also mandatorily be performed prior to ID to ensure implant integrity is sufficient prior to full compression.^24^ This was reported as mostly occurring in Australia but was not consistent practice (BSA 85%; 83% diagnostic). This again reflects a lack of understanding of the rationale for the non‐compressed series and the Eklund technique.

The results of this study reflect standard DBT use in the assessment setting only for BSA facilities and routinely, where available, for diagnostic sites (59%). The imaging series put forward by BSA respondents appears adjunct to the routine imaging series and by diagnostic respondents favouring the standard eight‐image Eklund series (31%). Similar to the results for 2D imaging, the remaining respondents reported a variety of different combinations of views, 5 and 11, for BSA and diagnostic imaging, respectively. The benefits of DBT in terms of lesion detection and the imaging of dense breasts are undeniable 41, 42, 43, 44, and its use should be implemented where possible.

The development protocols for imaging the augmented breast should consider the image acquisition system used and the benefits of separate imaging series for 2D and DBT technology. Further research into comparisons between dose and mammographic coverage of breast tissue between FFDM and DBT may contribute to a revised and standardised imaging series for use with this technology.

The standardisation of imaging techniques for both subglandular and submuscular breast implants, both of which have been shown to benefit from ID technique 45, 46, will contribute to uniformity of practice nationally and internationally. The introduction of uniform evidence‐based protocol will give mammographers the confidence that women are receiving the same standard of care regardless of where they present for imaging, with patients not receiving unnecessary imaging or radiation dose, or insufficient imaging, leading to poor mammographic coverage of the breast and potential missed pathology. In addition, the pursuit of an evidence‐based protocol reduces the risk of practices being liable if breast cancer goes undetected due to the lack of evidence supporting the varied imaging series currently used.

### Limitations

The study had several limitations, including a small number of respondents (*n* = 90). The method of survey distribution resulted in the exclusion of mammographers who are not members of the ASMIRT. Response rates also varied between questions, with a considerably smaller response rate to questions relating to DBT practice. This may be explained by its limited use in Australian diagnostic facilities and use only for assessment at BSA facilities.^47^ Potential recall bias may also result from the limited clinical exposure of some respondents to implant imaging. A lack of common language and understanding around the Eklund technique and ID imaging may also have impacted on responses.

## Conclusion

This study documents for the first time the imaging series used in contemporary imaging of the augmented breast in Australia. In this snapshot of current practice, the research has revealed that the eight‐view ID technique is the primary imaging series for both FFDM and DBT imaging of patients with breast implants The results of the study however also demonstrated varied additional imaging series used for routine imaging of patients with breast implants both between BSA and diagnostic imaging and within these providers. Little difference was demonstrated between imaging of breasts with submuscular and subglandular breast implants; imaging protocols are likely applied to all patients with breast implants rather than utilising a specific protocol based on implant position. Based on data obtained, current imaging is reportedly driven by dose reduction and radiologist preference within both facilities, possibly indicating an acceptance that sufficient mammographic coverage of the breast for breast cancer detection. The absolute amount of tissue imaged and that not included on the image during implant imaging remains unknown, as there is currently no evidence base upon which to draw. Looking forward the adoption of new technology such as coned beam breast CT may resolve many of these issues.

This research has identified the need for the establishment of dedicated evidence‐based imaging protocols for women with breast implants to ensure that regardless of which setting a woman attends that they will consistently receive an appropriate standard imagining series and imaging that results in sufficient mammographic coverage of their breast tissue for breast cancer detection and a dose that is ALARA. This is a reassurance that is not applicable to current practice. There is an urgent need for the development of evidence‐based standards to ensure that no woman is disadvantaged by imaging practice in Australia as a result of breast augmentation.

## Conflict of Interest

The authors declare no conflict of interest.
